# Liquid–Liquid Phase Separation and Assembly of Silk-like Proteins is Dependent on the Polymer Length

**DOI:** 10.1021/acs.biomac.2c00179

**Published:** 2022-07-07

**Authors:** Laura Lemetti, Alberto Scacchi, Yin Yin, Mengjie Shen, Markus B. Linder, Maria Sammalkorpi, A. Sesilja Aranko

**Affiliations:** † Department of Bioproducts and Biosystems, School of Chemical Engineering, Aalto University, Kemistintie 1, Espoo 02150, Finland; ‡ Academy of Finland Center of Excellence in Life-Inspired Hybrid Materials (LIBER), Aalto University, Kemistintie 1, Espoo 02150, Finland; § Department of Chemistry and Materials Science, School of Chemical Engineering, Aalto University, Kemistintie 1, Espoo 02150, Finland; ∥ Department of Applied Physics, School of Science, Aalto University, Otakaari 1, Espoo 02150, Finland; # Department of Bioproducts and Biosystems, Department of Chemistry and Materials Science, and Academy of Finland Center of Excellence in Life-Inspired Hybrid Materials (LIBER), School of Chemical Engineering, Aalto University, Espoo, 02150, Finland

## Abstract

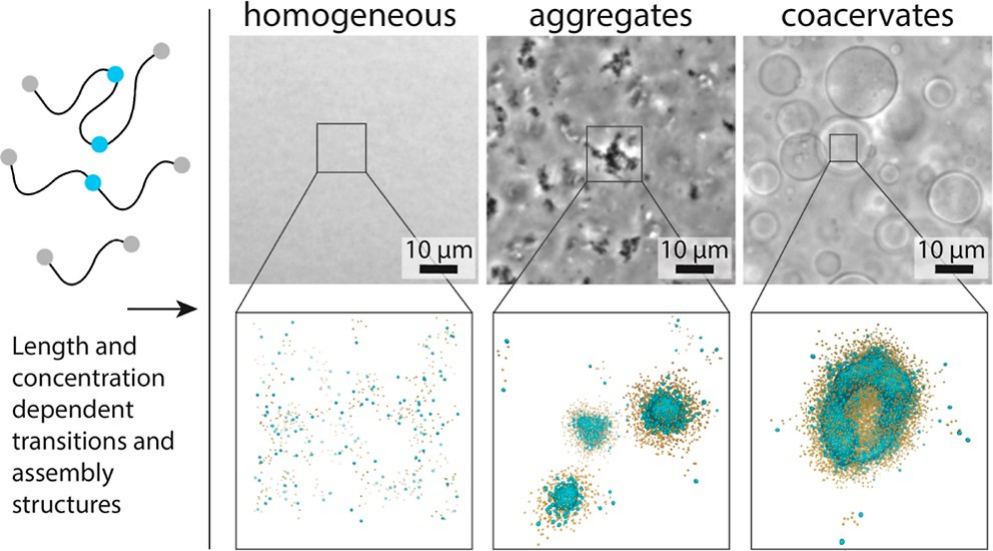

Phase transitions have an essential role in the assembly of nature’s protein-based materials into hierarchically organized structures, yet many of the underlying mechanisms and interactions remain to be resolved. A central question for designing proteins for materials is how the protein architecture and sequence affects the nature of the phase transitions and resulting assembly. In this work, we produced 82 kDa (1×), 143 kDa (2×), and 204 kDa (3×) silk-mimicking proteins by taking advantage of protein ligation by SpyCatcher/Tag protein-peptide pair. We show that the three silk proteins all undergo a phase transition from homogeneous solution to assembly formation. In the assembly phase, a length- and concentration-dependent transition between two distinct assembly morphologies, one forming aggregates and another coacervates, exists. The coacervates showed properties that were dependent on the protein size. Computational modeling of the proteins by a bead-spring model supports the experimental results and provides us a possible mechanistic origin for the assembly transitions based on architectures and interactions.

## Introduction

Coacervation plays a role in the initial molecular assembly steps of many natural materials with very diverse properties, like the underwater adhesives of mussels,^1^ the squid beak famous for the extreme stiffness gradient,^2^ and the elastin fibers in the human body.^3^ Coacervation enables spatiotemporally controlled preorganization and local concentration of the molecules and can further lead to liquid-to-solid transitions.^4,5^ Coacervation^6−8^ or formation of spherical droplets^9,10^ is a possible intermediate step also in spider silk fiber formation. Coacervation is observed when the protein solution separates into two immiscible liquid phases of low and high protein concentration. Both the phenomenon and the resulting coacervates are known to be strongly protein sequence and architecture (domain structure) dependent. It remains unclear how these govern the phase transition and the resulting assembly structures. Here, we focus on the effect of protein length using silk-mimicking protein constructs.

We have previously reported and characterized the assembly of engineered recombinant silk-like molecules consisting of an engineered spider silk-repeat sequence (eADF3) flanked by two cellulose-binding modules (CBMs), called CBM-eADF3-CBM.^6,17^ We found coacervation to be an essential intermediate step for the fiber formation of the silk-like molecule^6^ and that the coacervated solution could be used as an adhesive.^11,12^ The combination of computational and experimental data indicated that both the weak dimerization of the terminal CBM domains^13^ and weak interactions mediated by “sticker” regions in the repetitive middle part affect the assembly.^14^ The protein studied was 85 kDa in size, which is approximately only a third of the size of the native spider silks that have a size between 250 and 350 kDa.^15,16^ This prompted us to ask how extending the size close to that of the native spider silks would affect the assembly and phase transitions of the silk-mimicking proteins. Increasing the number of stickers typically promotes the formation of coacervates.^17,18−20^ The effect has, however, been studied with shorter proteins, in addition to which the effects of the weakly dimerizing terminal domains and the stickers in the repeat region made predicting the phase behavior nontrivial.

Unlike silkworm silk, spider silk cannot be produced by farming spiders due to their territorial and cannibalistic behavior,^21^ and therefore, we need to come up with ways to produce it recombinantly. Recombinantly produced silk proteins are, however, usually much shorter because the production yield of the large and repetitive silk proteins becomes very low in conventional expression hosts, such as*Escherichia coli.* Overcoming the size limitation by in vivo ligation has typically resulted in insoluble proteins, non-homogeneous samples, and/or low yields.^22,23^ Therefore, it is important to develop new biotechnological methods to produce native-sized silk proteins so that we would have a better starting point for studying the molecular assembly process.

In our current work, we approached the problem of recombinantly producing native-sized spider silk proteins by covalently conjugating shorter precursors in vitro, with the help of SpyCatcher2-SpyTag protein-peptide pair.^24,25^ SpyCatcher2 and SpyTag form a complex, which is covalently linked by an isopeptide bond that is autocatalytically formed between the side chains of a lysine in SpyCatcher and an aspartic acid in SpyTag. The reaction is fairly robust being able to endure diverse pH, temperature, and buffer conditions and yet leading to high yield.^25,26^ We produced the shorter precursors (66–77 kDa, depending on the terminal domains) in high yields in *E. coli*, followed by conjugation into 143 kDa (2×) and 204 kDa (3×) silk proteins in vitro. We focused on the characterization of the effect of the silk-protein’s length on the coacervation step. Gaining in-depth understanding on the coacervation process and their physical properties and molecular-level assembly is essential for understanding the assembly mechanisms of the silk proteins that is essential for the formation of silk materials. We defined the critical concentrations for the two assembly morphologies, aggregation and coacervation, and showed that they are dependent on the protein length. Two distinct assembly morphologies were also seen in computational modeling with coarse-grained bead-spring models of the 1 unit, 2 units, and 3 units long silks. The modeling provided us insights into the molecular-level interactions underlying the assembly phases.

## Experimental Section

### Cloning, Protein Expression, and Purification

**Figure 1:**
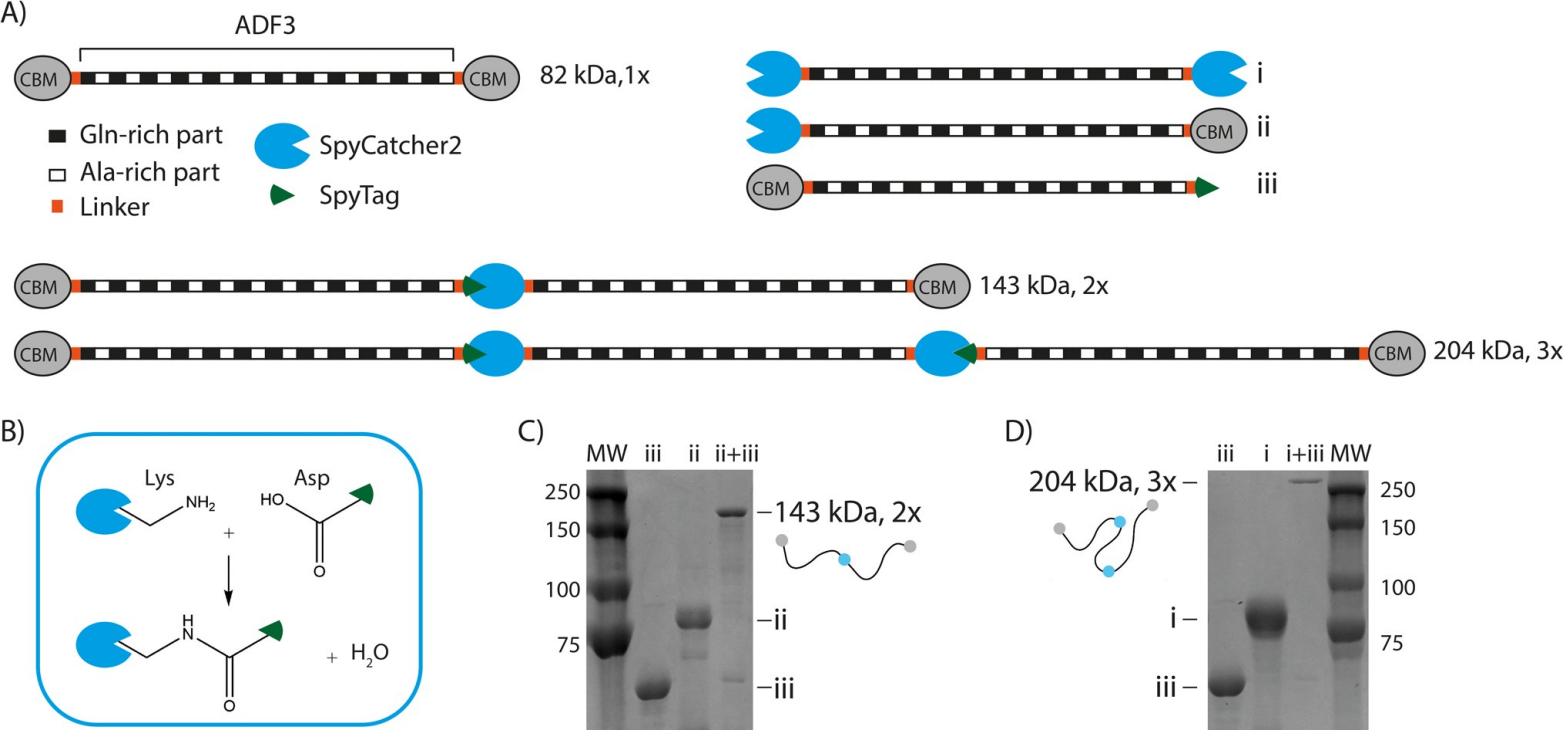
Schematic representation of the (A) precursors for longer proteins and the ligation products, (B) formation of the isopeptide bond, and (C,D) SDS-PAGE gels showing the ligation reaction to obtain 143 kDa (2×) (C) and 204 kDa (3×) (D) silks. MW stands for a molecular-weight marker.

The fusion proteins studied in our work have a triblock structure (Figure 1) similar to the ones reported previously.^6^ Part of the ADF3 dragline sequence from *Araneus diadematus* was used as the mid-block in all of them. All three terminal groups, CBM, SpyTag (ST), and SpyCatcher2 (SC2), were fused to the mid-block with short linkers (2 kDa). CBMs were obtained from *Clostridium thermocellum* cellulosome. The used constructs are CBM-ADF3-CBM, CBM-ADF3-ST, SC2-ADF3-CBM, and SC2-ADF3-SC2.

### Molecular Cloning

CBM-ADF3-CBM was obtained as a synthetic gene from GeneArt and inserted into the pEt28-vector between *Nco*I and *Xho*I restriction sites, resulting in the plasmid pSAEt42. To construct CBM-ADF3-ST (pSAEt56), the sequence coding for SpyTag was amplified from a synthetic gene using two primers (SA27: 5-ACAGAATTCTAGCTCCGCACATATTGTT and SA28: 5-TGCTCGAGTTTGGTCGGTTTGTATGC), digested using *Eco*RI and *Xho*I, and ligated into pSAEt42.

The sequence encoding for SpyCatcher2 was also obtained as a synthetic gene from GeneArt. The SpyCatcher2 sequence was amplified using primers (YY03F: CCTCCATGGGTGCAATGGTTACCACACT and YY03R: ACTGCTAGCGCTGGTATGTGCATCACCTTTGGTTGC) and digested using *Nco*I and *Nhe*I, and using primers (YY04F: TGCGAATTCTAGCTCCGCAATGGTTACCACACTGAGCG and YY04R: GTGCTCGAGACTGGTATGTGCATCACCTTTG) and digested using *Eco*RI and *Xho*I. SC2-ADF3-CBM (pYY3b) was constructed by replacing the N-terminal CBM domain of CBM-ADF3-CBM with the SpyCatcher2 domain using *Nco*I and *Nhe*I restriction sites. SC2-ADF3-SC2 (pYY3c) was constructed by replacing the C-terminal CBM domain in SC2-ADF3-CBM with SpyCatcher2 using *Eco*RI and *Xho*I restriction sites. Protein sequences are available in the Supporting Information.

### Protein Expression and Purification

Expression of each precursor and the control protein was carried out in EnPresso B500 media (EnPresso). Purification was carried out in two ways depending on the experiments where the protein was used. Precursors were purified by immobilized metal affinity chromatography (IMAC, ÄKTA purifier, GE Healthcare) for phase diagrams and fluorescence recovery after photobleaching (FRAP) experiments. Heat precipitation (CBM-ADF3-CBM 65 °C 20 min/CBM-ADF3-ST 65 °C, 30 min/SC2-ADF3-CBM 60 °C, and 20 min/SC2-ADF3-SC2 70 °C, 30 min) was used for inverse capillary velocity (ICV) experiments and scanning electron microscopy (SEM) images. For IMAC purification, a binding buffer containing 20 mM imidazole and 500 mM NaCl at pH 7.4 and an elution buffer with 500 mM imidazole and 500 mM NaCl at pH 7.4 were used. Proteins were transferred to Tris-buffer (50 mM Tris, 20 mM NaCl, pH 7.4) using EconoPac 10 DG columns (Bio-Rad).

### Preparation of Longer Silk Proteins

Ligation to obtain both double (143 kDa silk) and triple length (204 kDa silk) silk proteins were achieved by mixing their precursors in a correct ratio at room temperature in Tris-buffer (50 mM Tris HCl, 20 mM NaCl, pH 7.4). CBM-ADF3-ST and SC2-ADF3-CBM were the precursors for 143 kDa silk, and CBM-ADF3-ST and SC2-ADF3-SC2 were used to obtain 204 kDa silk. CBM-ADF3-CBM was used as a reference and is referred to as 82 kDa silk in this study.

Concentrations of the precursors were determined based on band intensities from Coomassie blue-stained SDS-PAGE gels using ImageJ software. Concentrations were determined by comparing the intensities to that of an IMAC-purified reference sample of which the concentration had been determined with amino acid analysis. The ratio giving the best yield was chosen based on the small-scale ligations, and this was used to carry out large-scale ligation. Ligation proceeded to almost its full extent within the first 10 min, but reactions were always carried out overnight to ensure the complete reaction. Ligation yields were analyzed from O/N samples from Coomassie-stained SDS-PAGE gels based on band intensities, using ImageJ (NIH), assuming that all proteins bind the stain equally. Three independent reactions for 143 kDa (2×) and 204 kDa (3×) silks were analyzed.

All proteins were concentrated to a sufficiently high concentration in order to induce liquid–liquid phase separation using 30 kDa molecular-weight cutoff centrifugal concentrators (Vivaspin, Sartorius). The approximate final concentration of silk proteins was determined, as described above. In addition, the presence of coacervates was verified with optical microscopy.

### Phase Diagrams

**Figure 2:**
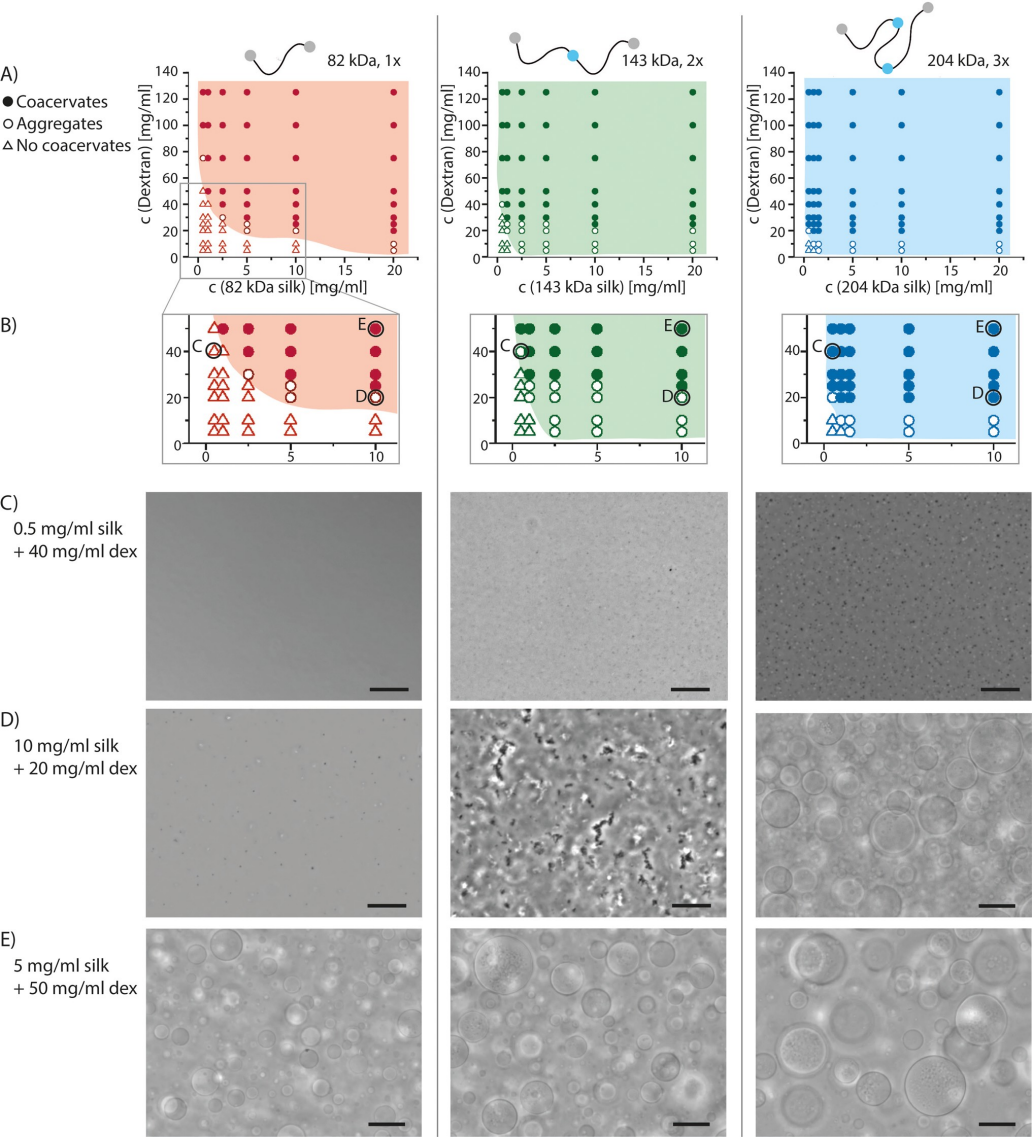
A) Phase diagrams of the silks with dextran (500 kDa). (B) Zoom-in from the phase transition region. Open triangles indicate homogeneous solution, open circles indicate aggregates, and filled circles indicate coacervates. The colored area in the phase diagram represents the two-phase region showing either aggregates or coacervates. (C–E) Optical microscopy pictures showing differences in phase separation behavior between silk proteins of different lengths. 82 kDa (1×) silk is shown on the left panels, 143 kDa (2×) silk in the middle panels, and 204 kDa (3×) silk on the right panels. Scale bars 20 μm.

Stock solutions of the 82 kDa (1×), 143 kDa (2×), and 204 kDa (3×) silks were prepared with different concentrations from 1 to 40 mg/mL and of dextran from 10 to 250 mg/mL. These solutions were mixed in a 1:1 ratio in order to reach the final concentrations, as shown in the phase diagrams in Figure 2. The solution was imaged with an optical microscope directly after mixing.

Two morphologically different phase-separated states were observed. The transitions between aggregated and coacervated morphologies of the 204 kDa (3×) silk were studied further. The coacervated sample was prepared by mixing silk protein with dextran in final concentrations 35 and 17.5 mg/mL, respectively, followed by imaging under the light microscope. The coacervated sample was then diluted with buffer (50 mM Tris, 20 mM NaCl, pH 7.4) in a small vial into the final concentration of 20 mg/mL silk protein and 10 mg/mL dextran, in which aggregates were formed, prior to observation under the microscope. To observe transition from aggregated morphology to coacervates, a fresh sample containing aggregates (20 mg/mL silk protein and 10 mg/mL dextran) was prepared and imaged. The sample was then mixed in a vial with dextran to end up in the final concentration of 15 mg/mL silk protein and 40 mg/mL dextran, followed by imaging the coacervates under the microscope.

### Labeling

The silk proteins were labelled with Oregon Green 488 (Thermo Fisher Scientific), a green-fluorescent dye, for FRAP experiments. Oregon Green 488 reacts with the amine group of Lys residues. IMAC-purified 82 kDa silk was labeled in deionized water, and the pH was adjusted to 8.3 with 1 M NaHCO_3_. Since one of the Lys residues in the SpyCatcher2 participates in the ligation with SpyTag, the ligation was carried out first in order to avoid affecting the ligation reaction. The reaction was carried out in 50 mM Tris HCl, 20 mM NaCl buffer, pH 8.3. To fluorescently label the proteins, approximately 0.5–0.7 mg of the dye was dissolved to dimethylformamide, and this solution was mixed with the protein solution. The reaction mixture was gently stirred while being protected from light for an hour after which unreacted dye was removed with EconoPac 10 DG desalting columns. All labeled proteins were purified by IMAC, either prior or after the labeling reaction, followed by buffer exchange with Econo-Pac 10 DG desalting columns to the Tris buffer.

### Fluorescence Recovery after Photobleaching (FRAP) and Confocal Microscopy

FRAP was conducted to determine the diffusion speed of a fluorescently labeled protein. Coacervated samples for FRAP were prepared by mixing the IMAC-purified fluorescently labelled protein with heat-purified protein, followed by concentrating the solution until phase separation was observed. Aggregated samples were prepared by mixing the same labeled sample with dextran, which allowed precisely adjusting the concentrations to ensure that the samples were in the aggregated region in the phase diagram. It was not possible to study the very small and highly mobile aggregates, but we could get data on the larger aggregates.

An area with 2 μm diameter inside a coacervate was photobleached with a focused laser beam, and the recovery of the fluorescence was recorded. This area corresponded to approximately 30% of the coacervate area. FRAP imaging was carried out using a Leica TSC SP5 confocal microscope with a FRAP booster equipped with a 63 × 1.2 NA water objective, argon laser (488 nm) and 488/561 dichroic beam splitter were used for imaging, and Leica AF Lite–TCS MP5 software together with Matlab was used to analyze the data. The data were fitted to eq 1(27)
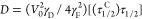
1where *V*_0_ is the known velocity of the calibration scan, *τ*_1/2_ and *τ*_1/2_^C^ are the 50% recovery times for a diffusion experiment and calibration scan, respectively, and *γ*_D_ and *γ*_F_ represent the shape of the beam and the extent of the bleaching, respectively. Diffusion constants are presented as mean values (with standard deviation). Fluorescence images showing the aggregated phase were taken from fresh samples containing aggregates using the Leica TSC SP5 confocal microscope.

### Inverse Capillary Velocity

ICV was determined from the fusion events of two coacervates, as reported by Brangwynne et al.^28^ For this, videos of fusion events were recorded with an Axio Vert.A1 inverted optical microscope equipped with an AxioCam 503 color camera (Carl Zeiss, Germany). Each fusion was then analyzed frame by frame to obtain the aspect ratio (AR) at each point in time. AR was determined by drawing an ellipse, in which the area corresponds to the area of the two droplets, and measuring the long and short axes of the ellipse, AR = *l*_long_/*l*_short_. ImageJ was used for analysis. The AR was then plotted versus time, and characteristic relaxation time τ was determined by fitting the following exponential function to the data
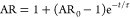
2where AR_0_ is the initial aspect ratio. The characteristic length *l* at the beginning of the fusion event was calculated according to eq 3
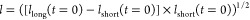
3

*τ* and characteristic length were then plotted for several events, and the ICV was determined from a linear fit to these data. Only *τ* values obtained from fits having *R*^2^ ≥ 0.99 were included.

### Optical Microscopy

Phase-separated samples were imaged with an Axio Vert.A1 inverted optical microscope equipped with an AxioCam 503 color camera (Carl Zeiss, Germany).

Protein and dextran samples for the phase diagrams were prepared beforehand in several different concentrations in 50 mM Tris HCl, pH 7.4 and, prior to imaging, mixed in a 1:1 ratio in order to reach the final concentrations, as shown in the diagrams.

### Scanning Electron Microscopy

Electron microscopy imaging was carried out with a Zeiss Sigma FE-SEM with variable pressure. A secondary electron detector and 1.5 kV EHT were used.

Aggregated morphologies were arrested by vitrification as follows. One droplet (20 μL) of the 204 kDa (3×) silk protein sample containing aggregates (40 mg/mL silk protein in 50 mM Tris and 20 mM NaCl, pH7.4) was plunged and vitrified in propane (cooled to its freezing points by thermal contact with liquid nitrogen). Samples were then handled under liquid nitrogen and transferred into a vacuum chamber for drying.

Film samples were prepared by pipetting 8 μL of silk solution on the parafilm and stretching the parafilm after the protein droplet had almost fully dried. Fiber samples were prepared by pulling the fiber from a concentrated silk dope with the help of tweezers. Samples were coated with 7 nm of platinum or platinum/palladium prior to imaging.

### Computational Modeling

Assembly of the modular CBM-ADF3-CBM and the SC2/ST-terminated block-protein constructs was computationally modeled by a coarse-grained bead-spring model, in which the terminal protein units were described by spherical beads A with diameter *σ*_AA_, and the secondary structure-induced “sticker regions”^14^ in the flexible ADF3 middle part were modeled by seven smaller spherical beads B with diameter *σ*_BB_ = *σ*_AA_/2. A sketch of the protein models is shown in Figure 8A. It is worth noting that both CBM and the SC2/ST ligation links were represented by bead A in the model, despite the differences in the constructs. Additionally, the model did not differentiate different “sticker regions” corresponding to the beads B, that is, the smaller beads model the presence of multiple effective “stickers” in the flexible part without addressing their nature or specific number. Consecutive spherical beads were connected via the potential
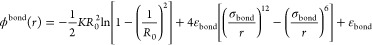
4

**Figure 3:**
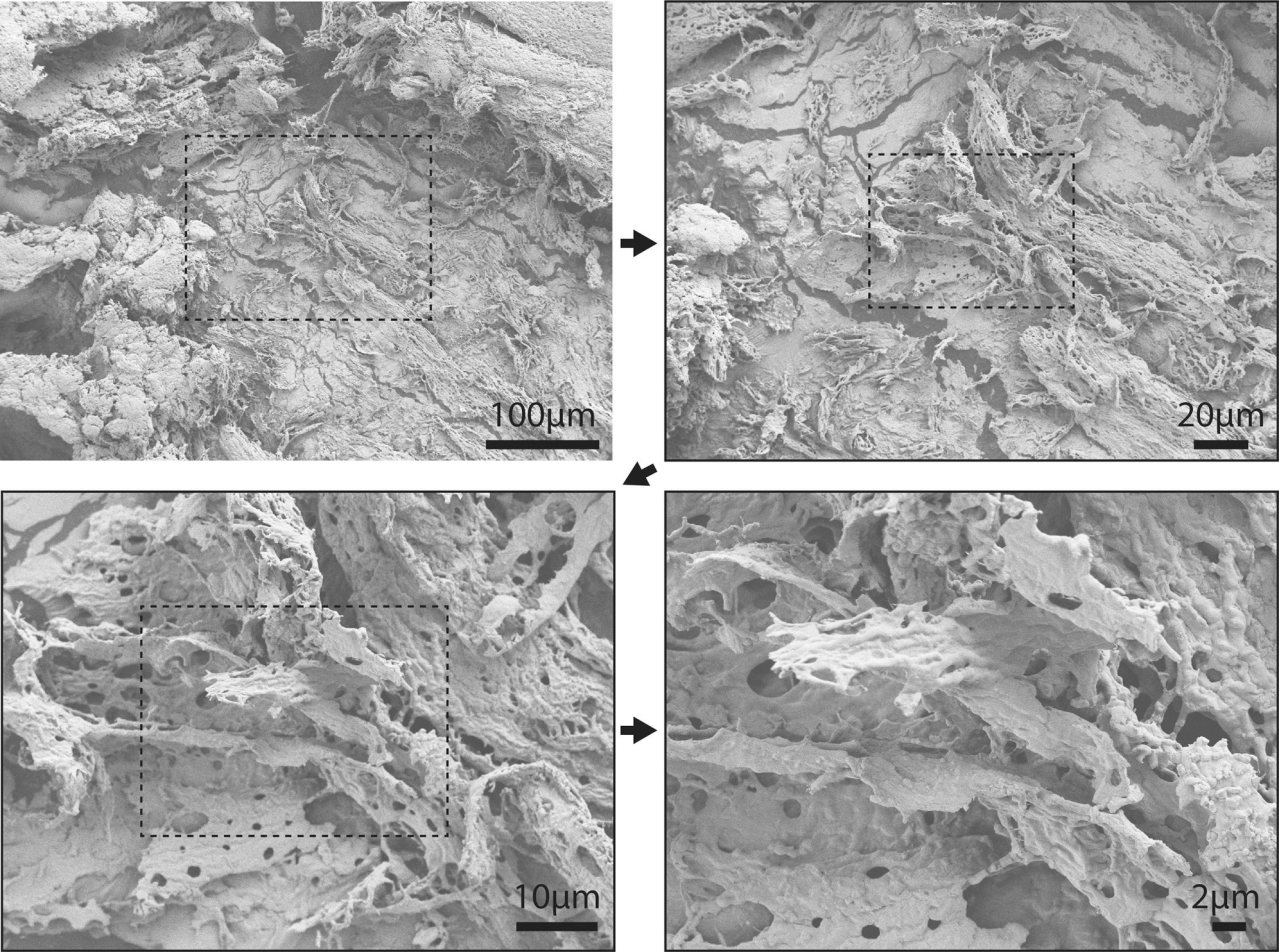
SEM images of the vitrified and freeze-dried aggregates of the 204 kDa (3×) silk.

**Figure 4:**
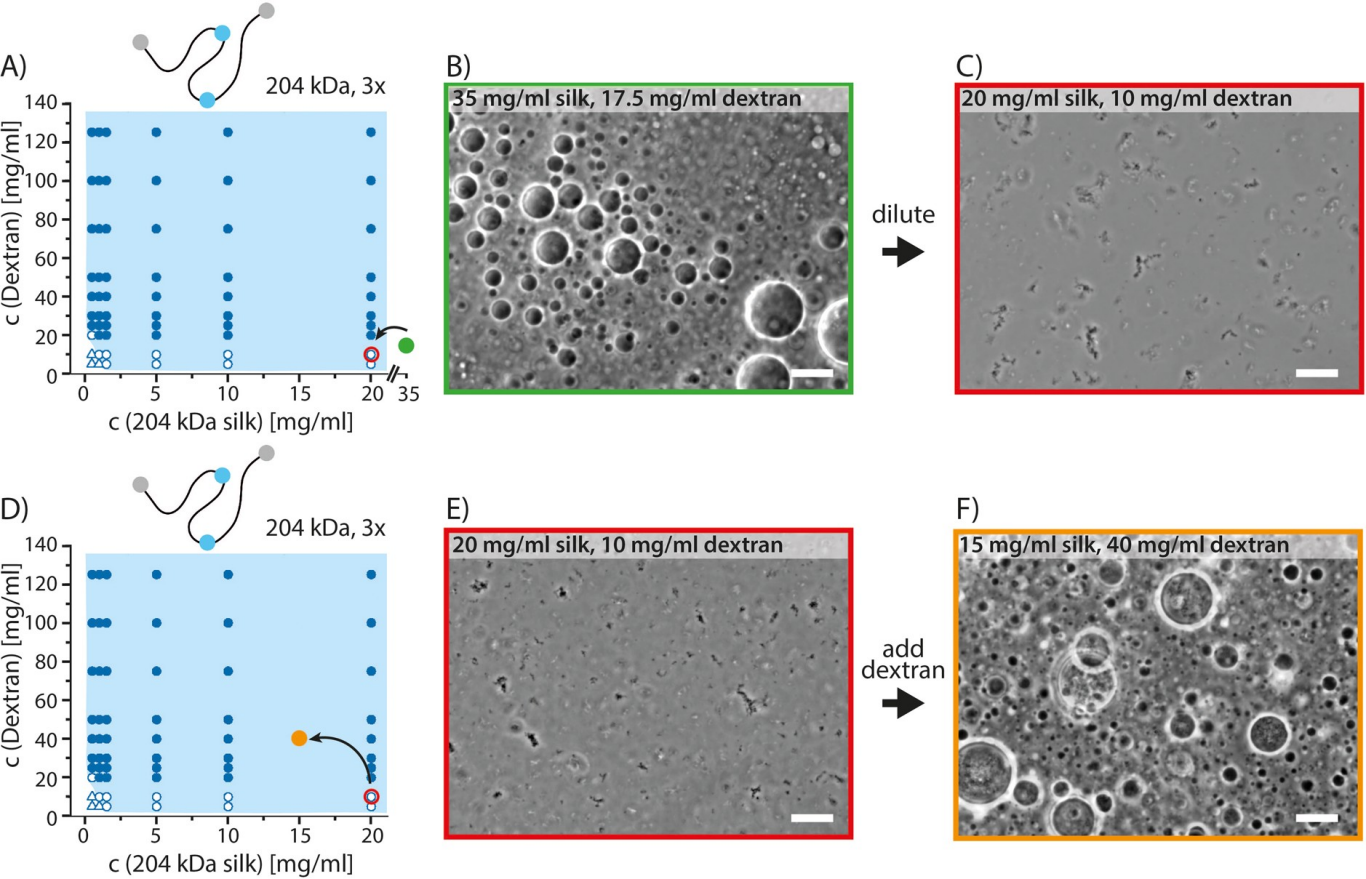
Transitions between coacervated and aggregated morphologies. Optical microscopy images of 204 kDa (3×) silk solution: (A) transition from the coacervate region to the aggregate region in the phase diagram. (B) Coacervated sample. (C) Aggregates formed after diluting the coacervated sample. (D) Transition from the aggregate region to the coacervate region in the phase diagram. (E) Aggregated solution. (F) Coacervates formed after addition of dextran to the aggregated sample. Scale bars 20 μm. Open triangles indicate homogeneous solution, open circles indicate aggregates, and filled circles indicate coacervates.

**Figure 5:**
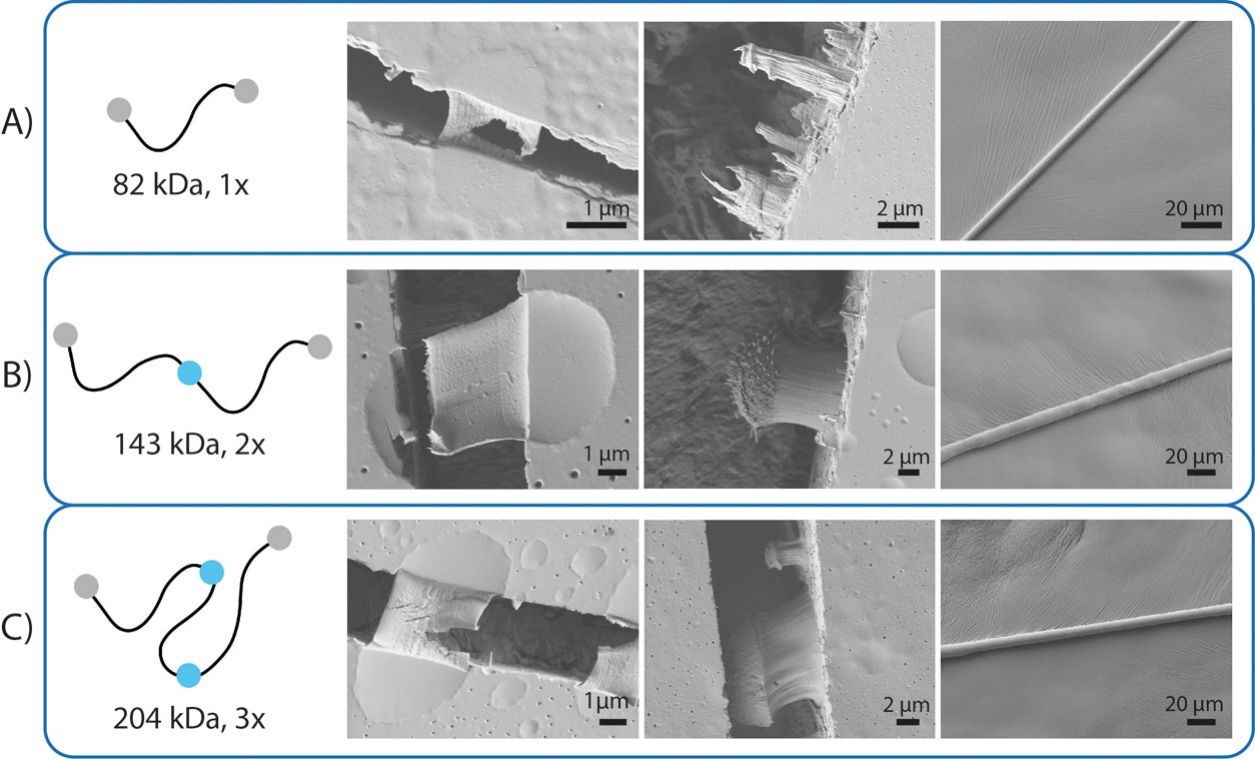
SEM images of (A) 82 kDa (1×) silk, (B) 143 kDa (2×) silk, and (C) 204 kDa (3×) silk.

**Figure 6:**
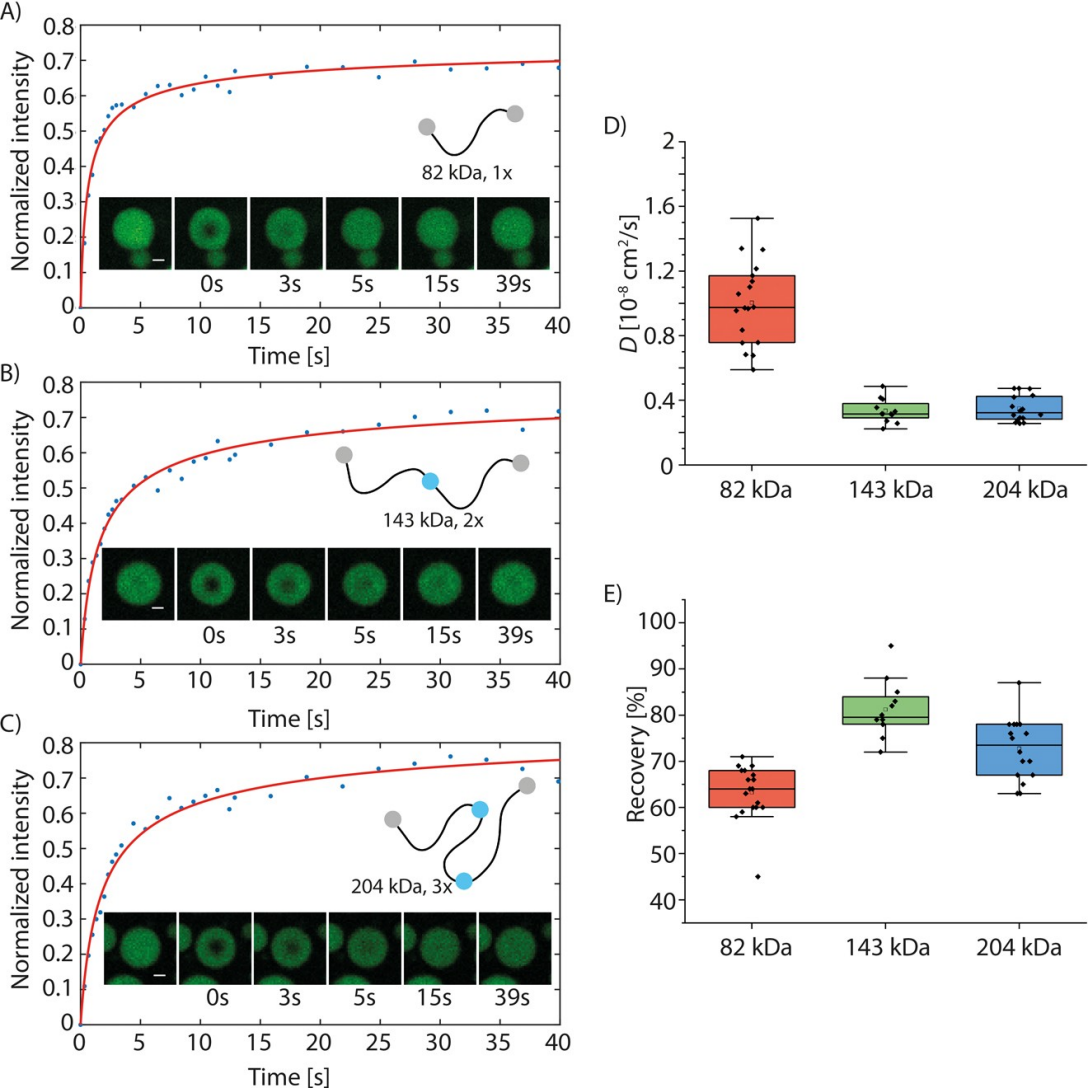
FRAP recovery of partially bleached silk protein coacervates for (A) 82 kDa (1×) silk, (B) 143 kDa (2×) silk, and (C) 204 kDa (3×) silk. Scale bar 2 μm. (D) Diffusion constant shown as box plots. Diffusion speed is presented as a mean value ± std, *n* = 18 for 82 kDa (1×) silk, *n* = 12 for 143 kDa (2×) silk, and *n* = 16 for 204 kDa (3×) silk. Here, *n* is the number of independent measurements. (E) Fluorescence recovery on the bleached area is shown as box plots.

**Figure 7:**
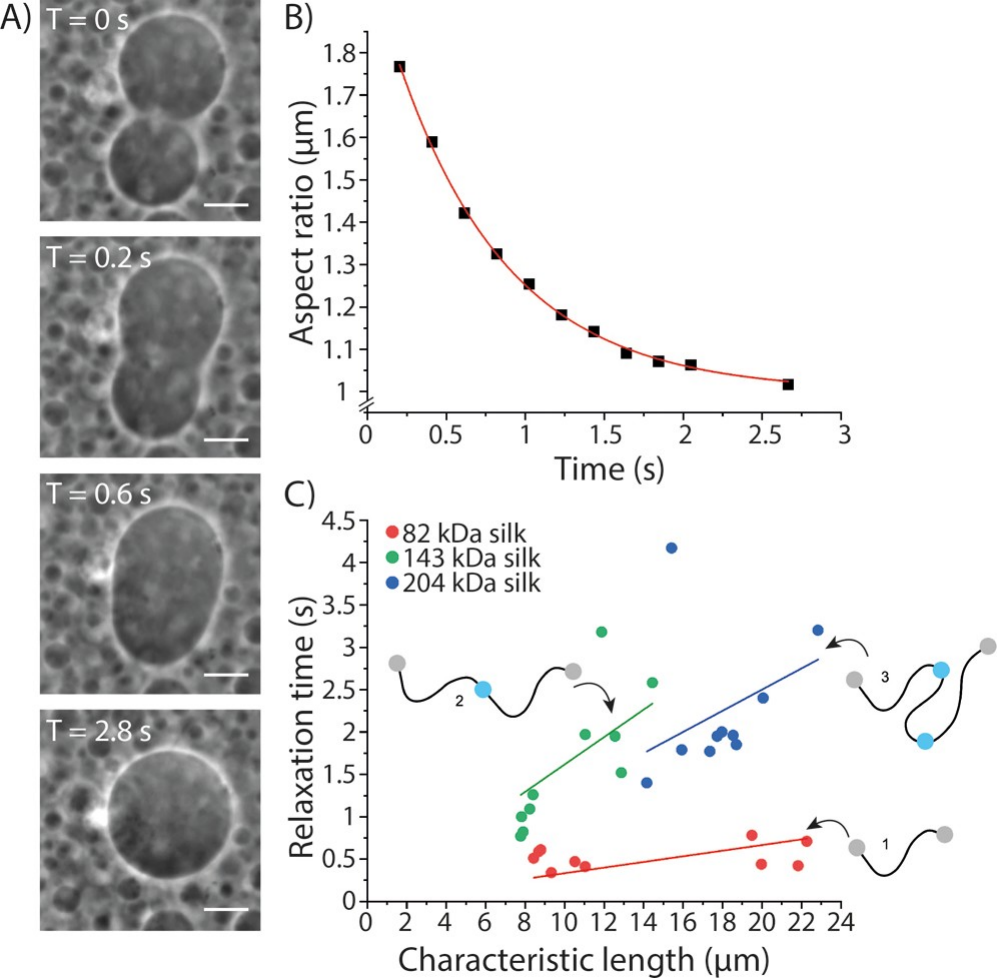
ICV analysis from fusion of coacervates. (A) Fusion of two coacervates of 82 kDa (1x) silk shown as an example. Scale bar 10 μm. (B) Development of the AR over time from the fusion shown in (A). (C) Plot of characteristic relaxation time, *τ*, vs characteristic length *l*, in which the lines represent linear fit through 0 to each data set.

**Figure 8:**
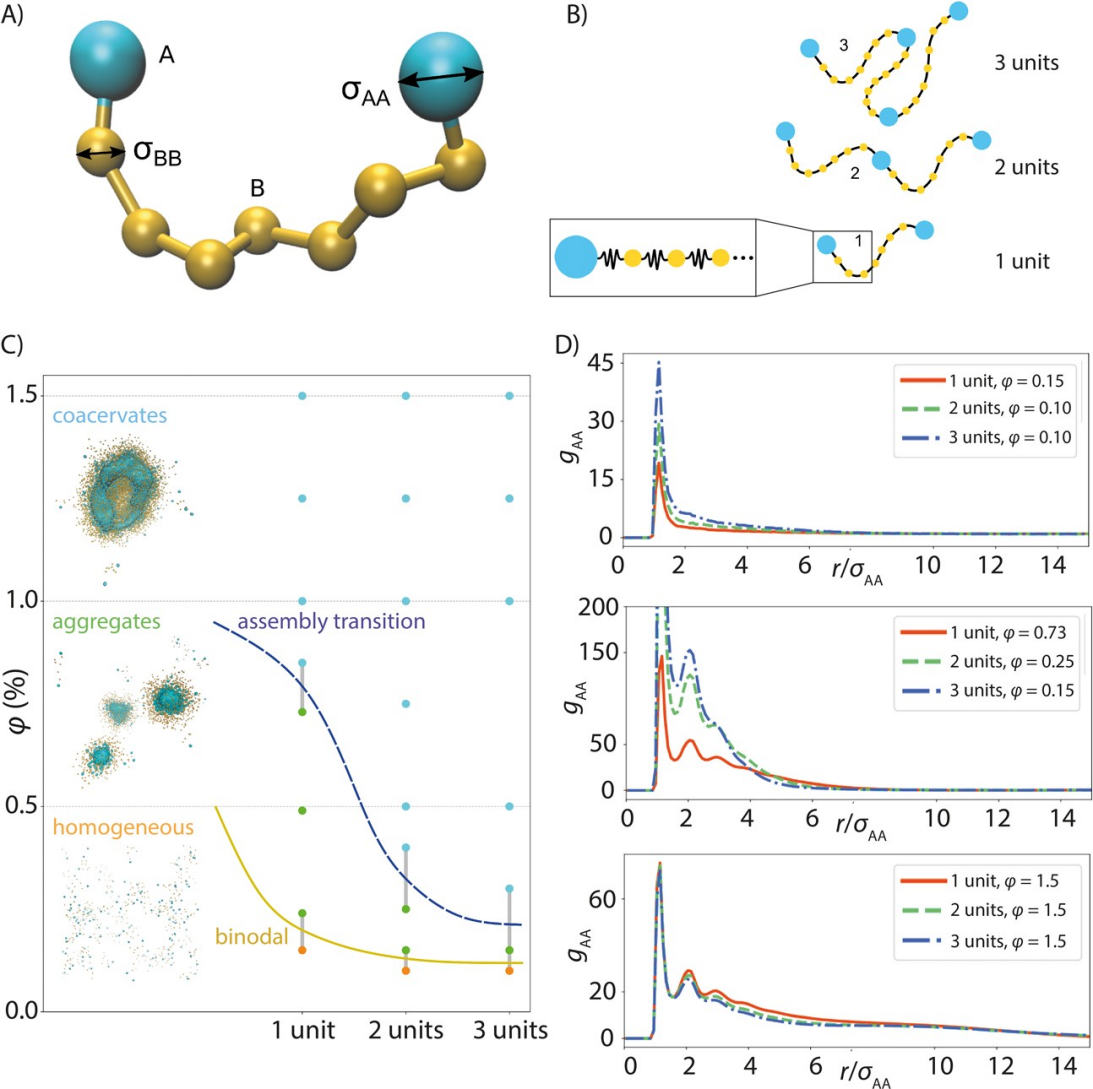
Computational model for the phase behavior of silk and assembly characterization by it. (A) Design and variables of the bead-spring model. (B) Schematic presentation of the bead-spring model of the 1 unit, 2 units, and 3 units long silks. (C) Computational assembly phase diagram for the simplified bead-spring protein models of 1 unit, 2 units, and 3 units in the simulations at different volume fractions *φ*. The visualizations show representative final simulation configurations for each of the assembly phases for the short 1 unit silk models. (D) Structural analysis of the assemblies via radial distribution function *g*_AA_(*r*) calculated for the end beads A in the assemblies corresponding to different volume fractions.

The first term is the standard finitely extensible nonlinear elastic (FENE) bond,^29,30^ with tether strength *βKσ*_AA_^2^ = 20 and range *R*_0_ = 3*σ*_AA_, where *β* = (*k*_B_*T*)^−1^. Here, *k*_B_ is the Boltzmann constant, and *T* is the temperature. Rest of eq 4 defines the minimal distance between consecutive beads via a truncated Lennard-Jones potential (Weeks-Chandler–Andersen potential), cutoff 2^1/6^*σ*_bond_. We set *σ*_bond_ = 2*σ*_AA_ and *βε*_bond_ = 1. Pairwise interactions were calculated using the standard Lennard-Jones potential
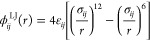
5

The *σ*_*ij*_ describes the size, and *ε_ij_* is the attraction parameter for beads of type *i*, *j* = A, B. Mixing rules *σ*_AB_ = (*σ*_AA_ + *σ*_BB_)/2 and *ε*_AB_ =  were used. A standard cutoff *r*_cut_ = 3*σ_ij_* was used for the interactions.^31^ Note that the interactions between consecutive beads were modeled only via eq 4, and the contribution from eq 5 was neglected.

Time evolution of the system was obtained based on the standard Brownian dynamics simulations approach. In this, the fundamental equations of motion correspond to the Langevin equation
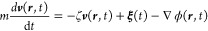
6at the overdamped limit
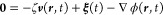
7

In our previous work,^32^ the overlap concentration of dextran was measured to be 16 g/L. This indicates that using the overdamped limit of Brownian dynamics provides a good approximation of the dynamics in terms of the viscosity response of the currently examined silk protein solution. In this, *ζ* is the friction, and the stochastic term ***ξ***(*t*) had a mean value *ξ*_*k*_(*t*) = 0 and a time correlation *ξ*_*k*_(*t*)*ξ*_l_(*t*^′^) = 2*D*_0_*δ*_*kl*_*δ*(*t* – *t*^′^) for *k*, *l* = *x*, *y*, and *z*, where *δ*_*kl*_ is the Kronecker delta-function and *δ*(*t* – *t*^′^) is the Dirac delta function. The last term −∇*ϕ*(**r**,*t*) represents the force due to interparticle interactions, here eqs 4 and 5. Time evolution was obtained by
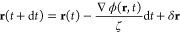
8where the three components of *δ***r** are sampled from a Gaussian distribution with standard deviation 2*D*_0_d*t*. The time scale of the simulations was set by the unitless quantity *τ*_s_ = *σ*_AA_^2^/*D*_0_, where *D*_0_ is the bare diffusion constant of the beads. The simulations were performed in scaled units such that *σ*_AA_ = *D*_0_ = 1.

The LAMMPS package^33^ was used for the simulations. The attraction parameters were set to *βε*_AA_ = 2 and *βε*_BB_ = 0.1, respectively. This choice imposes that association is mainly driven by the terminal units A, whereas the contributions by the middle beads B are less important, following the findings of ref (14). A cubic simulation box with edge length *L* = 40*σ*_AA_ was used. Equilibration time was 150 × 10^6^d*t*, where the time step d*t* = 5 × 10^–5^*τ*_s_, corresponding to a total equilibration time of 7500*τ*_s_. Consecutive production run was 100 × 10^6^d*t*, which was used for structural and average mean squared displacement analysis. The average diffusion constants *D* were obtained via linear regression performed over the second half, that is, 50 × 10^6^d*t*, of seven (12 for volume fraction *φ* = 1.5%) independent runs. An error estimate was obtained from standard deviation.

## Results and Discussion

We designed three variants of the 82 kDa triblock protein CBM-ADF3-CBM^6^ by replacing either one or both of the terminal CBM domains with either SpyCatcher2 or SpyTag (Figure 1A). Each of the resulting three precursors, CBM-ADF3-SpyTag (i), SpyCatcher2-ADF3-CBM (ii), and SpyCatcher2-ADF3-SpyCatcher2 (iii), contained a mid-block consisting of a 43 kDa fragment of ADF3 silk. Mixing SpyCatcher2 and SpyTag resulted in an autocatalytic formation of an isopeptide bond between the Catcher and Tag, resulting in a covalent ligation of the fusion proteins (Figure 1B).^25^ Mixing the precursors (ii) and (iii) or (i) and (iii) resulted in the covalent conjugation of 143 kDa (2×) and 204 kDa (3×) silk, respectively, as observed based on analysis of the proteins’ molecular weights by SDS-PAGE (Figure 1C,D). The reaction was typically completed within an hour with a high yield (Figures 1C,D and S1) and homogeneity. As expected, the yield from the reaction resulting into ligation of the 204 kDa (3×) silk was slightly lower than the yield for the 143 kDa (2×) silk because the latter only requires one conjugation step to take place.

The effect of the protein length on the onset of phase separation was studied with the help of the inert crowding agent dextran. We have previously shown that dextran does not interact with the protein, partition into the coacervates, or affect the functionality of the protein but only reduces the free volume.^32^ Dextran can therefore be used as a crowding agent, which enables constructing phase diagrams. All three silk constructs of different lengths, the 82 kDa (1× silk), 143 kDa (2×), and 204 kDa (3×) silk, formed coacervates above certain concentration threshold (Figure 2). The critical concentration for the coacervation to occur was the lower the longer the protein was. A solution with the 82 kDa (1×) silk at concentration of 0.5 mg/mL mixed with 40 mg/mL dextran remained clear, whereas the 204 kDa (3×) silk in the same conditions formed coacervates (Figure 2B,C). To exclude that the properties of SpyCatcher2 and/or SpyTag could cause the observed effect, we also defined phase diagrams of the precursors alone as a control experiment (Figure S2). The data confirmed that the precursors containing SpyCatcher2 and SpyTag were not more prone to coacervation but had an opposite effect, further highlighting the effect of the length of the silk region. At high enough concentrations, such as 10 mg/mL silk and 50 mg/mL dextran, all the constructs coacervated (Figure 2B,E). The coacervates formed by the 143 kDa (2×) and 204 kDa (3×) silks were liquid-like and readily fused together, similar to what was previously observed for the 82 kDa (1×) construct.^6^

In addition to coacervation, also another morphology was observed for all the silk constructs of different lengths (Figures 2–4 and S2–S5). Assemblies with irregular shapes were observed at concentrations slightly below those required for the coacervation to occur. We call these assembly aggregates to distinguish them from the liquid-like spherical coacervates. The aggregates remained in solution and are not referring to irreversible aggregation involving a macroscopic liquid-to-solid transition^34^ but to an assembly morphology.^35^ The longer, 143 kDa (2×) and 204 kDa (3×), silks resulted in more wide aggregate regions in the phase diagrams. At lower protein and dextran concentrations, both aggregates and coacervates were very small but still easily visually distinguished from each other due to the irregular shape of aggregates and round shape of coacervates. Aggregates could be observed also without dextran (Figure S4). SEM images of the vitrified and freeze-dried aggregates showed μm-scale fiber-like structures (Figure 3).

We studied the reversibility of the aggregated and coacervated morphologies of 204 kDa (3×) silk under the light microscope (Figure 4). We first moved from the coacervated region in the phase diagram into the aggregate region (Figure 4A) by diluting a coacervated sample (Figure 4B) with buffer (50 mM Tris, 20 mM NaCl, pH 7.4). In the diluted sample, only aggregates and no coacervates were visible (Figure 4C). We then tested whether we could move from the aggregated region into the coacervated region (Figure 4D). An aggregated sample (Figure 4E) was pushed into the coacervated region by adding dextran. The coacervates formed within the time it took to mix and image the sample (Figure 4F). No qualitative difference was observed in the aggregated samples prepared either directly from the homogeneous stock solution or via the coacervated phase (Figure 4C,E). We observed that in addition to the small reversible aggregates, there were also larger aggregates, which became more prominent and stable when the samples aged (data not shown). These larger aggregates could still be seen in the coacervated sample both within and outside the coacervates, whereas the smaller aggregates could not be observed after the coacervates formed (Figure 4F). The data indicate that both the aggregates and the coacervates are reversible.

We have previously reported that coacervation is an important intermediate step for fiber formation for recombinant silk proteins.^32^ Here, we wanted to investigate whether the longer constructs would retain the tendency to form fiber structures. We studied thin films of the protein solutions by SEM, in which the coacervates were easily seen as spherical regions (Figure 5). Small fibers protruding from the coacervates were observed by SEM in films that had been stretched to fracture in a semidried state. We observed clusters of fibers emerging from broken coacervates on the surface of the film and additionally large bundles of fibers protruding from inside the film at the fractured edge. Furthermore, fibers could be pulled from concentrated and coacervated silk dopes of all the silk length variants, 82 kDa (1×), 143 kDa (2×), and 204 kDa (3×), with the help of tweezers (Figure 5). These data show that the longer silk variants have similar key functionalities than the shorter silk-mimicking proteins.^6^

The first SEM image in each row presents the microfibrillar structures that arise from a coacervate when a semi dry protein film is stretched. The second SEM image shows variations in microfibrillar structures arising when the film was stretched until it cracked. The images at the right show SEM images of the fibers drawn from each silk length. Since it was evident from the phase diagrams that the length of the protein affects the phase behavior, the properties of the coacervates were further characterized. The diffusion inside the coacervates was studied with FRAP. The results confirm that in all cases the coacervates are liquid like as the fluorescence recovers relatively fast (Figure 6A–C). As expected, diffusion of the 82 kDa (1×) silk (*D* = 1.00 ± 0.25 × 10^–8^ cm^2^/s) in the coacervates was faster than 143 kDa (2×) and 204 kDa (3×) silks (*D* = 0.33 ± 0.07 × 10^–8^ cm^2^/s and *D* = 0.35 ± 0.08 × 10^–8^ cm^2^/s, respectively) (Figure 6D). Interestingly, the diffusion rates of the 143 kDa (2×) and 204 kDa (3×) silks in the coacervates were almost identical. The recovery of the 82 kDa (1×) silk was lower (63%) than that of the 143 kDa (2×) (81%) and 204 kDa (3×) (74%) silks (Figure 6E). The faster diffusion rate of the shorter proteins is presumably leading to the bleaching of larger number of the proteins and thus lower recovery ratio. It is worth noting that thermodynamic phase co–existence equilibrium leads to the protein concentration in condensates for each silk length to be independent of dextran addition, that is, available total volume or change in the total protein concentration. This is because the chemical potential difference between the dilute and condensed phase can be assumed to remain constant under identical experimental conditions.

We also examined the diffusion of the molecules in the aggregates. Based on light microscopy, the aggregates had irregular shapes but could form and deform in the reversible manner (Figures 2–4). We studied the aggregates formed by the longest, 204 kDa (3×), silk proteins by FRAP (Figure S5). It was not possible to capture the diffusion in the smallest and highly mobile aggregates, but we were able to follow the recovery of the bleached area in some of the larger and more stable aggregates in three different protein concentrations (1% dextran and 3.3/10/20 mg/mL of protein) (Figure S5A,B). Due to the small and irregular size of the aggregates, it was not possible to distinguish between the recovery observed due to molecules moving in the dilute phase from the recovery of the fluorescence within the aggregates. Visual examination does, however, reveal that there is partial recovery even in the larger aggregates studied (Figure S5B). Although the observed recovery is remarkably slower than that within the coacervates (Figure 6), it indicates that the proteins in the aggregates have mobility.

Since FRAP data suggest that the diffusion rate inside the coacervates is similar for the 143 kDa (2×) and 204 kDa (3×) silks, we further studied the ICV of the coacervates of different silk lengths. ICV was determined from videos taken from the fusion events of two coacervates (Figure 7). The slope of the linear fit, as shown in Figure 7C, gives us the ICV *η*/*γ* where *η* is the viscosity of the droplet and *γ* is the surface tension. The results from ICV showed a similar trend to those observed with FRAP experiments. The 82 kDa (1×) silk had the faster ICV of 0.033 ± 0.005 s/μm compared to the longer silks, whereas the ICV of the 143 kDa (2×) and the 204 kDa (3×) silks was similar, with ICV values 0.162 ± 0.016 and 0.125 ± 0.014 s/μm, respectively. The results of the FRAP and ICV experiments showing similar mobilities for the 143 and 204 kDa silks may indicate that after overcoming certain threshold in the polymer length, the polymer entanglement does not increase anymore. We have previously carefully characterized the coacervates formed by the 82 kDa (1× silk) to demonstrate that they are liquid-like and shown by Fourier transform infrared spectroscopy that the silk proteins within the liquid-like coacervates were rich in *α*-helical conformations and did not undergo conformational transition to *β*-sheets under these experimental conditions.^6^ The reversibility of the coacervated phase (Figure 4A,B), the high mobility observed in the FRAP measurements (Figure 6), and the ability to fuse by the droplets studied in the ICV experiments (Figure 7) strongly indicate that also the 143 kDa (2×) and 204 kDa (3×) silks form liquid-like coacervates, and no aggregation or other large conformational transitions take place.

To obtain more insights into the assembly response behind the phase diagrams of Figure 2, we used a computational modeling approach with a simplified bead-spring model to approximate the protein constructs. In the modeling, the examined variables were protein length and volume fraction of protein in the system. The latter, in addition to capturing increasing protein concentration, effectively also models dextran addition as the crowding agent reduces the free volume available to the protein solution. This means that the dilute vs. condensate phase separation occurs in a smaller total volume when dextran is added. Figure 8 presents the assembly phase diagram and corresponding representative assembly structures. The simulation-based phase diagram is showing a homogeneous solution at a low concentration (small volume fraction) of proteins, an assembly transition to aggregates with increasing concentrations with an even higher volume fraction leading to another, possibly kinetic transition. Consistent with the experimental phase diagrams (Figure 2), also the simulations indicated that a smaller protein concentration is sufficient for assembly formation when the products are longer. Increasing length also facilitated the second transition. This can be understood in terms of increasing the total attraction strength with the increasing protein chain length, see for example ref (36).

Notably, a simple Lennard-Jones fluid where the particles have a fixed attraction strength (above the critical value) transitions from a homogeneous phase to an assembly phase by going over the binodal curve when increasing the concentration. Crossing the binodal signifies phase separation via nucleation: in both the experiments and the simulations, we see this transition. Further away from the binodal curve, we observe another transition, again in experiments and simulations. This transition rises from the kinetic character of the assembly changing by the effective assembly barrier decreasing such that the assembly no longer occurs via the same pathway as that leading to the “aggregates.”

Let us next examine more in detail the structure of the assemblies in the aggregate and the coacervate regions in the simulations. Figure 8 presents also structural analysis of the assemblies with radial distribution function *g*_AA_(*r*) data corresponding to the distribution of the terminal beads A. The *g*_AA_(*r*) data show that even in the homogeneous solvation phase, the terminal beads associated (showed positional correlations), but as the phase remained a homogeneous solution, this did not emerge as the longer-range order. The finding is consistent with observations of Fedorov et al.^13^ where weak dimerization of the CBM terminal units, with *K*_D_ of 90 ± 30 μM was reported. In the aggregate phase, it is notable that the terminal beads showed significantly stronger clustering, that is, positional correlations persisting over a distance separation corresponding to several terminal bead diameters *σ*_AA_. Here, the protein chain length affected the assembly structure with the longer protein constructs being able to pack in a more correlated way. Notable difference to the aggregate phase was that in the coacervate phase, the structural order persisted over length scales, characteristic to the simulation system size. Additionally, here the proteins exhibited some degree of correlations, regardless of the chain length. This arose from the ordered aggregates transitioning in the coacervate formation range to assemblies, in which the terminal units formed a percolating-like structure that dynamically reorganizes during simulation. The structure and its fluid-like character are in line with previous atomistic detail modeling^14^ and cryo-SEM results.^6^

**Figure 9:**
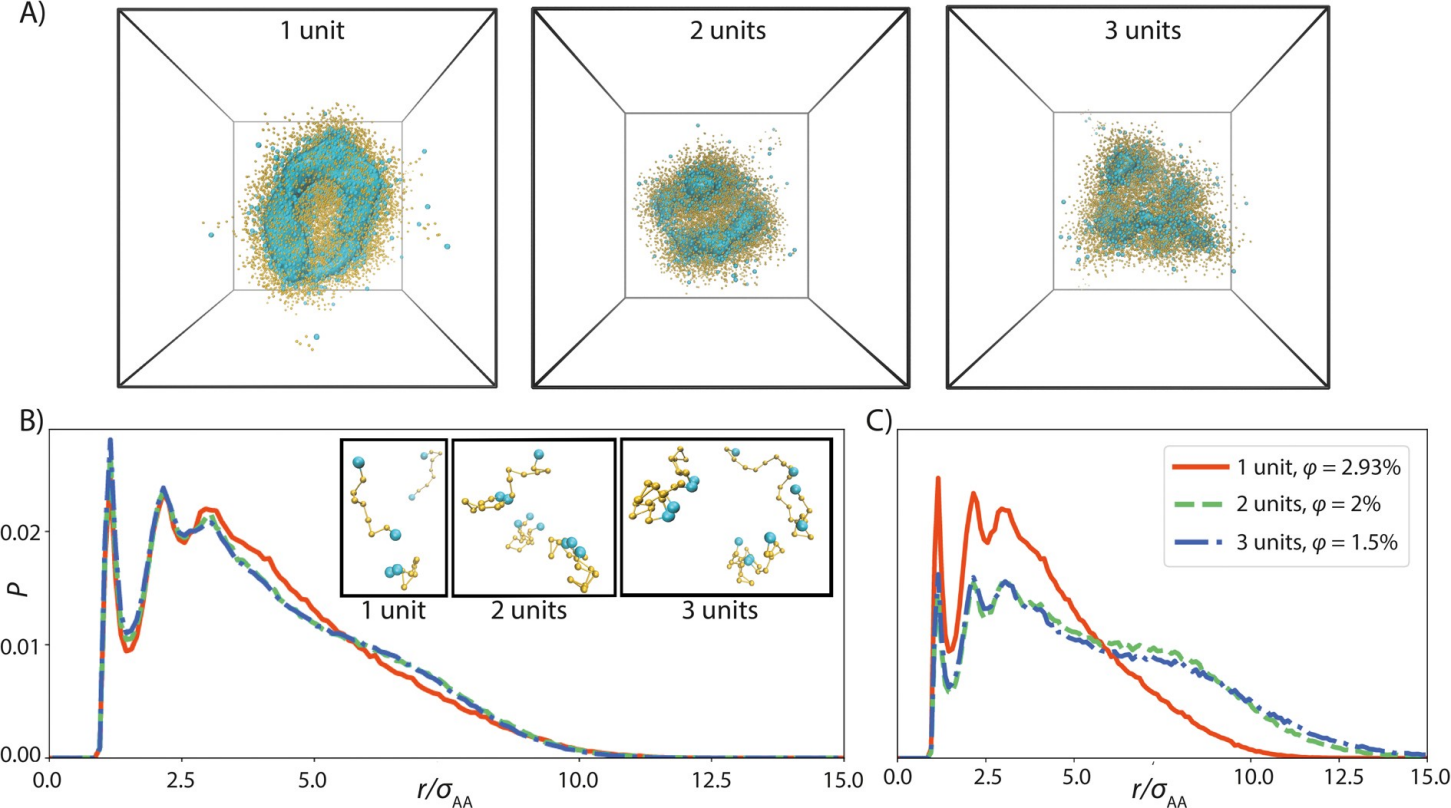
Structural analysis of the coacervate assembly. (A) Visualizations of representative snapshots of configurations adopted by the models for 1 unit, 2 units, and 3 units long silk models at *φ* = 2.93, 2.0, and 1.5% volume fractions, respectively. (B,C) Structural analysis of the coacervate assemblies for 1 unit, 2 units, and 3 units silk models. The graphs present probability distribution *P* of distance between consecutive A beads in the protein chains (B) and protein chain end-to-end A beads (C) in the models. The 3 units data correspond to averaging over three independent runs for improved statistics.

Analysis of the protein conformations in the assemblies (Figure 9) revealed that in the region associated with coacervates, the consecutive A beads in the 1 unit, 2 units, and 3 units long silk models adopted a very similarly correlated assembly structure. However, the length-dependent differences in the coacervate structure were revealed by the end-to-end packing of the silk models: namely, the 2 units and 3 units long silk models that contained in total three or four A beads in the models exhibit both intramolecular A bead pairing and extended configurations that spanned a connection network to neighboring proteins. The short 1 unit silk model had only two terminal A beads, which resulted in a preference of loop-like protein configurations. The configuration snapshots in Figure 9 visualize this. Notably, the extended, unpaired A beads could pair with neighboring proteins, forming a load carrying network.

**Figure 10:**
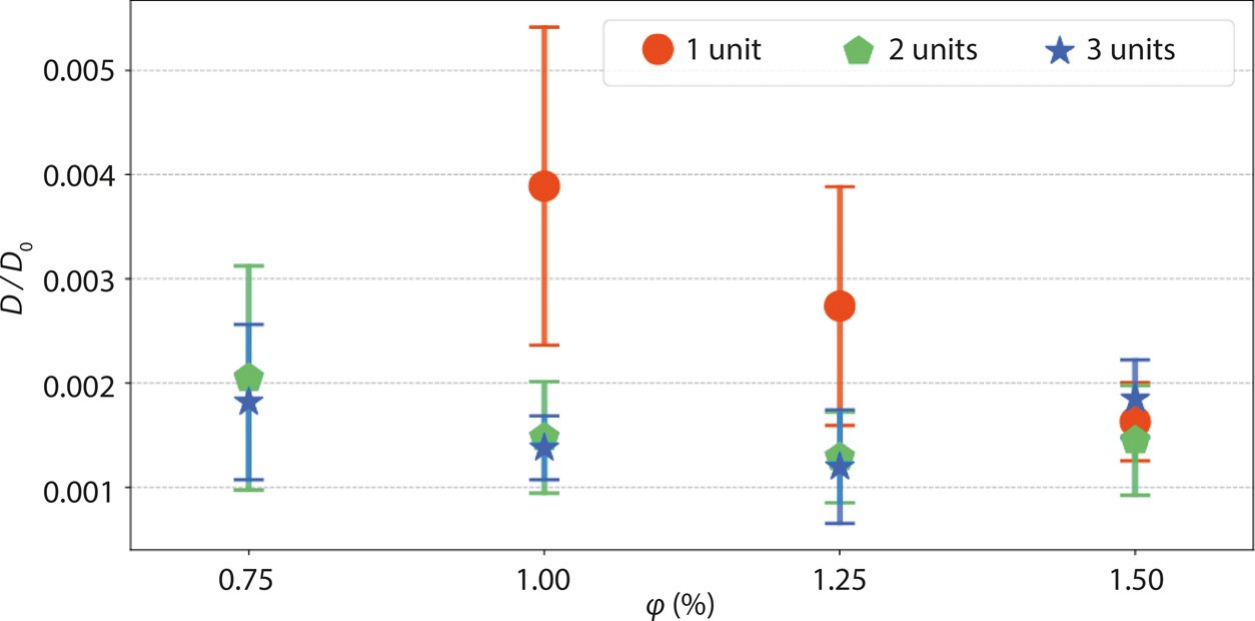
Relative diffusion coefficient of the proteins in simulations at different volume fractions. The normalization factor *D*_0_ is the dilute solution self-diffusion coefficient of single beads in the protein model.

Analysis of the simulation systems in terms of *D* revealed (Figure 10) that the diffusion is not only sensitive to the protein concentration (volume fraction) but also protein length. In the volume fraction range examined in the simulations, the short protein diffused significantly faster than the two longer constructs. This can be understood considering Figure 9, where the data show that the 2 units and 3 units long silk models (2× and 3×) adopted similar end-to-end configurations, whereas shorter 1 unit silk model (1×) packed more compactly, with the end bead positions strongly correlated. Similar conformational changes could be behind the experimental data (Figure 6). One should, however, not directly compare the relative diffusion coefficients extracted from the simulations to the diffusion in experiments: the simulation data shows that diffusion is clearly strongly concentration dependent and also non-monotonous in terms of concentration response.

As a final note on the simulation results, computational work has addressed the effect of protein sequence in coacervate formation also in prior studies. While polymer physics approaches provide guidelines and, for example, mechanism differences,^37−39^ at a coarse-grained molecular modeling level, Statt et al.^40^ mapped a number of phases arising from differences in sequence via a model, in which the “sticker” sequence varied. Rana et al.^35^ characterized also the effect of the protein chain length versus “stickers” in the proteins and predict ranges for finite size aggregates versus macroscopic phase separation using a Grand Canonical lattice Monte Carlo model. Our observations here using a model qualitatively matched with the experimental setup are consistent. However, the diffusion differences in the experiments and in the assembly results by our flexible bead-spring type model point toward folding and dynamics in the assembly being important in the silk-like protein system examined here.

## Conclusions

In this work, we examined the protein architecture dependency of assembly phases using three engineered spider silk-mimicking proteins of varying lengths. Solutions of all protein constructs not only showed phase separation to assemblies above a critical concentration but also emergence of two different kinds of assemblies both in experiments and simulations. Close to critical concentration, aggregates were formed while at higher concentrations, all systems showed a second assembly transition, now to formation of coacervates. The irregularly shaped aggregates were clearly distinct from the spherical coacervates. In the assembly response, not only the length of the proteins had a very strong effect on the transition positions, with longer silk constructs systematically pushing the critical concentration, but also the second transition to coacervates, to occur at a lower concentration.

We speculate that the aggregate versus coacervate transition corresponds to a decreasing effective barrier against assembly growth via diffusion or coalescence of the assemblies. The kinetics of the assembly differs in the systems with aggregates growing significantly slower, both in experiments and in simulations, than the condensate droplets (data not shown). Related to phase transitions of analogous macromolecular systems, we expect the protein system to be approaching the spinodal curve with increasing concentrations, see for example ref (41). It is interesting to consider whether the dynamics change associated with the transition bears spinodal characteristics already, but the current data set, both simulations and experiments, is insufficient to conclude the latter.

We presented here a characterization of the phase separation response of a model silk-like protein construct as a function of its length (1 unit vs. 2 units vs. 3 units) reporting two assembly morphologies with well-defined transitions governed by the concentration and a systematic and strong length dependency on the assembly structure. Significantly, more complex assemblies and the internal structure of the assemblies than observed here have been reported for biological condensates.^42,43^ The significance of the current work is that by mapping the phase separation response and characterization of the phases in terms of the structure, steps toward bottom-up design of protein materials based on sequence and block construct architecture are taken.

## Supporting Information Available

Protein sequences; Figures S1–S5: yields of the conjugation reactions, phase diagrams of CBM-ADF3-SpyTag and SpyCatcher2-ADF3-SpyCatcher2, confocal microscopy images of the aggregates, light microscopy images of the aggregates formed without dextran, and FRAP data of the aggregated morphology (PDF)
