# Supplementary material for: Liquid–Liquid Phase Separation and Assembly of Silk-like Proteins is Dependent on the Polymer Length
Source: Biomacromolecules. 2022 Jul 7;23(8):3142–53. doi: 10.1021/acs.biomac.2c00179 (PMC9364312; doi:10.1021/acs.biomac.2c00179)
Supplement: Supplementary file 1 — bm2c00179_si_001.pdf [file bm2c00179_si_001.pdf]

Supporting Information

The liquid-liquid phase separation and assembly of silk-like proteins is dependent on the polymer length

Laura Lemetti[a,b], Alberto Scacchi[b,c,d], Yin Yin[a,b], Mengjie Shen[a,b], Markus B. Linder[a,b], Maria Sammalkorpi[a,b,c*], A. Sesilja Aranko[a,b*]

[a]*Department of Bioproducts and Biosystems, School of Chemical Engineering, Aalto University, Kemistintie 1, 02150 Espoo, Finland*

[b]*Academy of Finland Center of Excellence in Life-Inspired Hybrid Materials (LIBER), Aalto University, Kemistintie 1, 02150 Espoo, Finland*

[c]*Department of Chemistry and Materials Science, School of Chemical Engineering, Aalto University, Kemistintie 1, 02150 Espoo, Finland*

[d]*Department of Applied Physics, School of Science, Aalto University, Otakaari 1, 02150 Espoo, Finland*

*corresponding author

Email addresses: maria.sammalkorpi@aalto.fi, sesilja.aranko@aalto.fi

Protein sequences of the silk-mimicking constructs used.

CBM-ADF3-CBM:

MGNLKVEFYNSNPSDTTNSINPQFKVTNTGSSAIDLSKLTLRYYYTVDGQKDQTFWCDHAAI
IGSNGSYNGITSNVKGTFVKMSSSTNNADTYLEISFTGGTLEPGAHVQIQGRFAKNDWSNYT
QSNDYSFKSASQFVEWDQVTAYLNGVLVWGKEPSASASASAGASAAASAGAGAGAGPGQQ
GPGQQGPGQQGPYGPGASAAAAAAGGYGPGSGQQGPSQQGPGQQGPGGQGPYGPGASAAA
AAAGGYGPGSGQQGPGGQGPYGPGSSAAAAAAGGNGPGSGQQGAGQQGPGQQGPGGSAA
AAAAGGYGPGSGQQGPGQQGPGGQGPYGPGASAAAAAAGGYGPGSGQGPGQQGPGGQGP
YGPGASAAAAAAGGYGPGSGQQGPGQQGPGQQGPGGQGPYGPGASAAAAAAGGYGPGYG
QQGPGQQGPGGQGPYGPGASAASAASGGYGPGSGQQGPGQQGPGGQGPYGPGASAAAAAA
GGYGPGSGQQGPGQQGPGQQGPGQQGPGGQGPYGPGASAAAAAAGGYGPGSGQQGPGQQ
GPGQQGPGQQGPGQQGPGQQGPGQQGPGQQGPGQQGPGGQGAYGPGASAAAGAAGGYGP
GSGQQGPGQQGPGQQGPGQQGPGQQGPGQQGPGQQGPGQQGPYGPGASAAAAAAGGYGP
GSGQQGPGQQGPGQQGPGGQASASASAAASAASTVANSSSNLKVEFYNSNPSDTTNSINPQF
KVTNTGSSAIDLSKLTLRYYYTVDGQKDQTFWCDHAAIIGSNGSYNGITSNVKGTFVKMSSS
TNNADTYLEISFTGGTLEPGAHVQIQGRFAKNDWSNYTQSNDYSFKSASQFVEWDQVTAYL
NGVLVWGKELEHHHHHH

CBM-ADF3-SpyTag:

MGNLKVEFYNSNPSDTTNSINPQFKVTNTGSSAIDLSKLTLRYYYTVDGQKDQTFWCDHAAI
IGSNGSYNGITSNVKGTFVKMSSSTNNADTYLEISFTGGTLEPGAHVQIQGRFAKNDWSNYT
QSNDYSFKSASQFVEWDQVTAYLNGVLVWGKEPSASASASAGASAAASAGAGAGAGPGQQ
GPGQQGPGQQGPYGPGASAAAAAAGGYGPGSGQQGPSQQGPGQQGPGGQGPYGPGASAAA
AAAGGYGPGSGQQGPGGQGPYGPGSSAAAAAAGGNGPGSGQQGAGQQGPGQQGPGGSAA
AAAAGGYGPGSGQQGPGQQGPGGQGPYGPGASAAAAAAGGYGPGSGQGPGQQGPGGQGP
YGPGASAAAAAAGGYGPGSGQQGPGQQGPGQQGPGGQGPYGPGASAAAAAAGGYGPGYG
QQGPGQQGPGGQGPYGPGASAASAASGGYGPGSGQQGPGQQGPGGQGPYGPGASAAAAAA

GGYGPGSGQQGPGQQGPGQQGPGQQGPGGQGPYGPGASAAAAAAGGYGPGSGQQGPGQQ
GPGQQGPGQQGPGQQGPGQQGPGQQGPGQQGPGQQGPGGQGAYGPGASAAAGAAGGYGP
GSGQQGPGQQGPGQQGPGQQGPGQQGPGQQGPGQQGPGQQGPYGPGASAAAAAAGGYGP
GSGQQGPGQQGPGQQGPGGQASASASAAASAASTVANSSSAHIVMVDAYKPTKLEHHHHH
H

SpyCatcher2-ADF3-CBM:

MGAMVTTLSGLSGEQGPSGDMTTEEDSATHIKFSKRDEDGRELAGATMELRDSSGKTISTWI
SDGHVKDFYLYPGKYTFVETAAPDGYEVATAITFTVNEQGQVTVNGEATKGDAHTSASASA
SAGASAAASAGAGAGAGPGQQGPGQQGPGQQGPYGPGASAAAAAAGGYGPGSGQQGPSQ
QGPGQQGPGGQGPYGPGASAAAAAAGGYGPGSGQQGPGGQGPYGPGSSAAAAAAGGNGP
GSGQQGAGQQGPGQQGPGGSAAAAAAGGYGPGSGQQGPGQQGPGGQGPYGPGASAAAAA
AGGYGPGSGQGPGQQGPGGQGPYGPGASAAAAAAGGYGPGSGQQGPGQQGPGQQGPGGQ
GPYGPGASAAAAAAGGYGPGYGQQGPGQQGPGGQGPYGPGASAASAASGGYGPGSGQQGP
GQQGPGGQGPYGPGASAAAAAAGGYGPGSGQQGPGQQGPGQQGPGQQGPGGQGPYGPGA
SAAAAAAGGYGPGSGQQGPGQQGPGQQGPGQQGPGQQGPGQQGPGQQGPGQQGPGQQGP
GGQGAYGPGASAAAGAAGGYGPGSGQQGPGQQGPGQQGPGQQGPGQQGPGQQGPGQQGP
GQQGPYGPGASAAAAAAGGYGPGSGQQGPGQQGPGQQGPGGQASASASAAASAASTVANS
SSNLKVEFYNSNPSDTTNSINPQFKVTNTGSSAIDLSKLTLRYYYTVDGQKDQTFWCDHAAII
GSNGSYNGITSNVKGTFVKMSSSTNNADTYLEISFTGGTLEPGAHVQIQGRFAKNDWSNYTQ
SNDYSFKSASQFVEWDQVTAYLNGVLVWGKELEHHHHHH

SpyCatcher2-ADF3-SpyCatcher2:

MGAMVTTLSGLSGEQGPSGDMTTEEDSATHIKFSKRDEDGRELAGATMELRDSSGKTISTWI
SDGHVKDFYLYPGKYTFVETAAPDGYEVATAITFTVNEQGQVTVNGEATKGDAHTSASASA
SAGASAAASAGAGAGAGPGQQGPGQQGPGQQGPYGPGASAAAAAAGGYGPGSGQQGPSQ
QGPGQQGPGGQGPYGPGASAAAAAAGGYGPGSGQQGPGGQGPYGPGSSAAAAAAGGNGP
GSGQQGAGQQGPGQQGPGGSAAAAAAGGYGPGSGQQGPGQQGPGGQGPYGPGASAAAAA
AGGYGPGSGQGPGQQGPGGQGPYGPGASAAAAAAGGYGPGSGQQGPGQQGPGQQGPGGQ
GPYGPGASAAAAAAGGYGPGYGQQGPGQQGPGGQGPYGPGASAASAASGGYGPGSGQQGP
GQQGPGGQGPYGPGASAAAAAAGGYGPGSGQQGPGQQGPGQQGPGQQGPGGQGPYGPGA
SAAAAAAGGYGPGSGQQGPGQQGPGQQGPGQQGPGQQGPGQQGPGQQGPGQQGPGQQGP
GGQGAYGPGASAAAGAAGGYGPGSGQQGPGQQGPGQQGPGQQGPGQQGPGQQGPGQQGP
GQQGPYGPGASAAAAAAGGYGPGSGQQGPGQQGPGQQGPGGQASASASAAASAASTVANS
SSAMVTTLSGLSGEQGPSGDMTTEEDSATHIKFSKRDEDGRELAGATMELRDSSGKTISTWIS
DGHVKDFYLYPGKYTFVETAAPDGYEVATAITFTVNEQGQVTVNGEATKGDAHTSLEHHHH
HH

Supplementary Figures

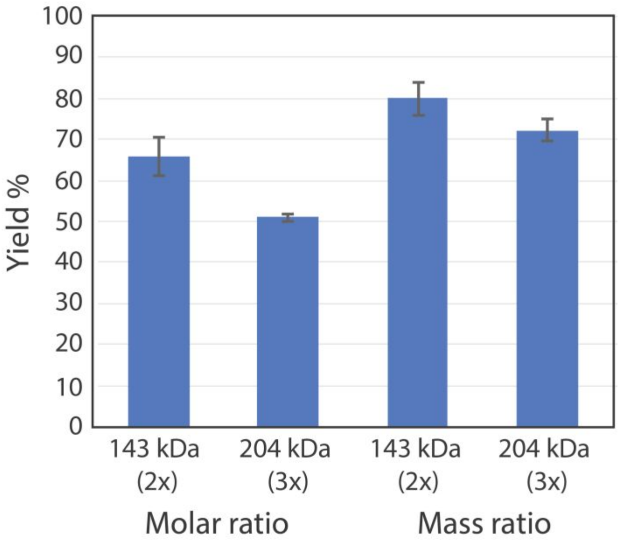

Figure S1. Yields of the conjugation reactions. Mean values ± std are shown, n=3.

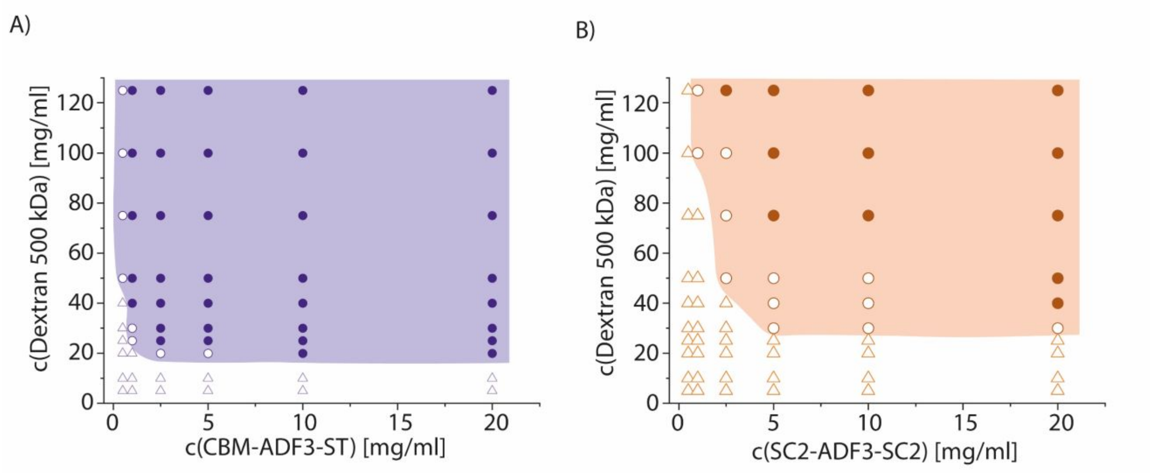

Figure S2. Phase diagrams showing the effect of concentration on the assembly morphology of A) CBM-ADF3-SpyTag and B) SpyCatcher2-ADF3-SpyCatcher2. Open triangles indicate clear solution, open circles aggregates, and filled circles coacervates. ST stands for SpyTag and SC2 for SpyCatcher2.

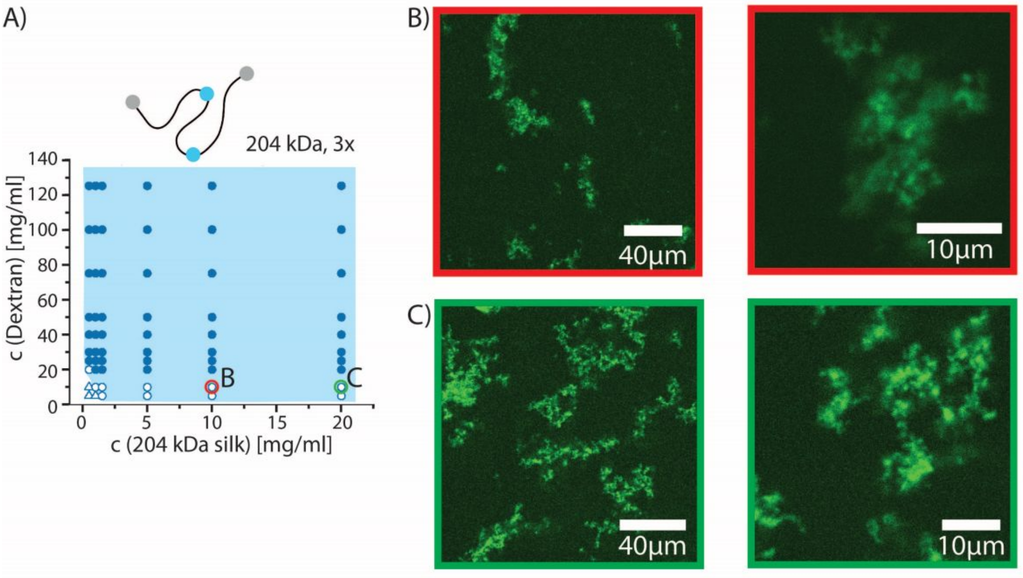

Figure S3. Confocal microscopy images of the aggregates. A) Phase diagram showing the region the samples were taken. B) Confocal microscopy images of the aggregates formed by the fluorescently labelled 204 kDa (3x) silk proteins.

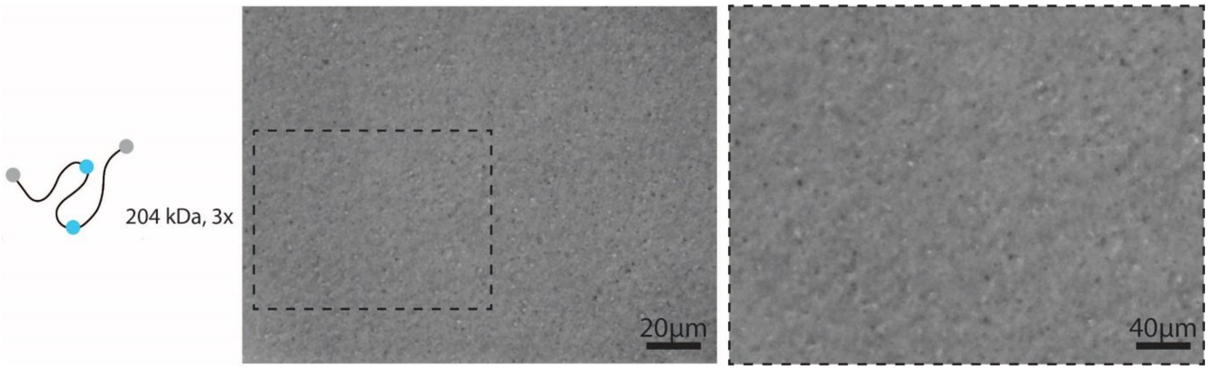

Figure S4. A light microscopy image of aggregates in sample containing 204 kDa (3x) silk protein in 40 mg/mL concentration (without dextran).

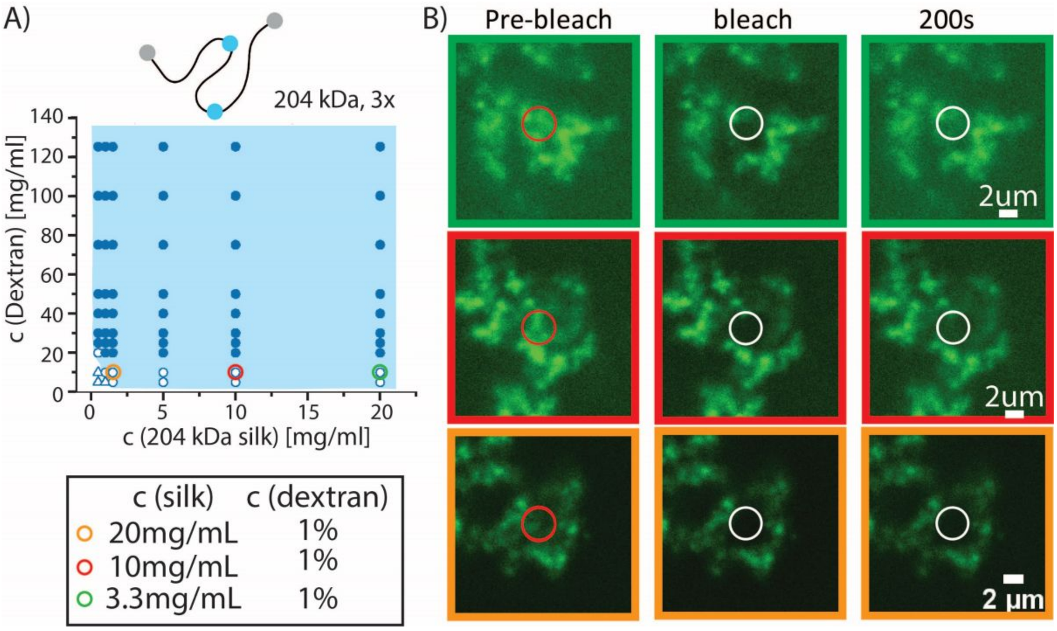

Figure S5. FRAP data of the aggregated morphology. A) Phase diagram showing the region the samples were taken. B) Confocal microscopy images of the aggregates formed by the fluorescently labelled 204 kDa (3x) silk proteins before bleaching, right after bleaching (0 s) and after recovering for 200 s. The red and white circles are showing the bleached region.
